# Informing the treatment of social anxiety disorder with computational and neuroimaging data

**DOI:** 10.1093/psyrad/kkae010

**Published:** 2024-05-03

**Authors:** Aamir Sohail, Lei Zhang

**Affiliations:** Centre for Human Brain Health, School of Psychology, University of Birmingham, Birmingham, B15 2TT, UK; Centre for Human Brain Health, School of Psychology, University of Birmingham, Birmingham, B15 2TT, UK; Institute for Mental Health, School of Psychology, University of Birmingham, Birmingham, B15 2TT, UK

## Social anxiety disorder through the lens of computational psychiatry

Current treatments for mental health disorders often demonstrate limited efficacy, stemming in part from a mismatch between a complex pathophysiology and the rudimentary categorical method of assessment and diagnosis (Kendler *et al*., 2011; Fried, 2022). Many researchers (in basic research and clinical research alike) have recently advocated for mental health disorders to be re-defined on the basis of computational principles and psychological constructs that more accurately map cognitive processes than dimensional approaches (Huys *et al*., 2021; Hitchcock *et al*., 2022). As a result, the burgeoning field of 'computational psychiatry' (Montague *et al*., 2012; Friston *et al*., 2014; Adams *et al*., 2016; Huys *et al*., 2016; Zhang, 2023) aims to do so by taking an interdisciplinary approach, incorporating facets of psychiatry, neuroscience, mathematics, and artificial intelligence. Most computational psychiatry research to date is theory driven (Huys *et al*., 2016; Hauser *et al*., 2022), allowing a formal account of mental health to be made by analysing how alterations related to disorders influence behaviour across various tiers of brain structures. This is implemented through an abductive strategy by proposing a normal functioning model and then altering it to generate new hypotheses for biological dysfunction, or using a deductive method, beginning with established neurobiological deficits observed in mental illnesses and incorporating these deficits into a computational model (Khaleghi *et al*., 2022). Theory-driven approaches have identified the computations underlying atypical behaviour for a range of mental health disorders including obsessive–compulsive disorders (Loosen & Hauser, 2020), autism spectrum disorders (Crawley & Zhang *et al*., 2020), schizophrenia (Kreis *et al*., 2022, 2023); attention deficit hyperactivity disorders (Ging-Jehli *et al*., 2021), psychopathy (Pauli & Lockwood, 2023), addiction (Kulkarni *et al*., 2023), and anxiety (Goldway *et al*., 2023).

Among anxiety, social anxiety disorder (SAD) represents a domain-specific instance, in which the core features of a negative self-view and a fear of negative evaluation may lead to the avoidance of social situations (Carleton *et al*., 2011; Clark *et al*., 2005). These behavioural and psychological changes arise from several differences in terms of the computational processing of social information. First, individuals with SAD demonstrate significantly higher learning from negative social feedback regarding the self (Koban *et al*., 2017), due to reduced uncertainty about self-positive attributes (Hopkins *et al*., 2021; Hoffmann *et al*., 2023). Such biased learning leads to the continued avoidance of social situations while affecting one's memory for socially valent information. For example, individuals with SAD demonstrating poorer memory for positive social experiences (Romano *et al*., 2020), as well as a greater negativity bias for perceived memories of social feedback (Johnston *et al*., 2023).

## Neurocomputational mechanisms of social feedback processing in SAD

**Figure 1:**
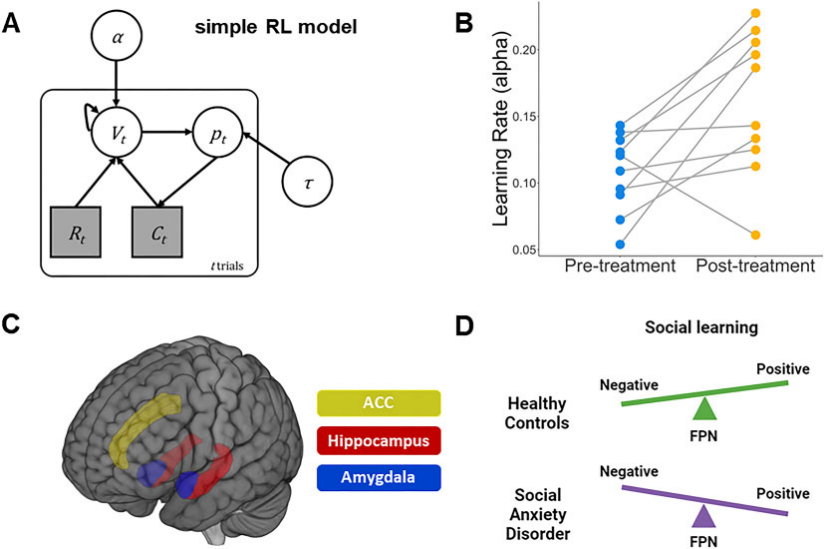
How computational and neuroimaging data can facilitate the treatment of SAD. (**A**) Illustration of a simple reinforcement learning (RL) model. α: learning rate; τ: choice stochasticity; V: action value; p: action probability; C: choice; R: outcome. (**B**) Computational models represent strong candidates as potential biomarkers, able to accurately capture parameter values indicative of treatment responsivity at the subject level. (**C**) Neuroimaging data of specific brain regions implicated with the pathophysiology of SAD can measure and predict patient responsivity to treatments. ACC: anterior cingulate cortex. (**D**) In the FPN model, social learning in response to feedback is modulated by the FPN, which is biased towards negative compared to positive social feedback in SAD. Adapted from Koban *et al*., 2023.

While previous studies demonstrate biased processing of social information in SAD, the brain regions orchestrating these biases were not well known. In a recent publication, Koban *et al*. (2023) used a functional magnetic resonance imaging (fMRI) paradigm in which healthy volunteers (*n* = 16) and individuals with SAD (*n* = 16) were asked to give a speech about their ideal job, with self-evaluation and self-esteem measured in response to positive and negative feedback received from an ostensible interview panel. The study employed reinforcement learning model to uncover social learning biases (Fig. 1), and mediation-based fMRI analyses to uncover direct and indirect effects between feedback mismatch, brain activity, and changes in self-perception.

The results first replicated their earlier findings (Koban *et al*., 2017), highlighting the increased tendency of those with SAD to learn from negative feedback compared to neurotypical individuals. Using fMRI, the authors demonstrated that this bias was mediated by the anterior insula/frontal operculum, with the ventromedial prefrontal cortex buffering social influence effects on changes in self-evaluation. Further analyses implied a top-down regulatory role of these regions by the frontoparietal network (FPN), a network proposed to modulate top-down regulatory role of self-related content (Dixon & Gross, 2021). The authors ultimately suggest a theoretical model in which FPN responses are negatively biased in SAD, leading to more negative social learning (Fig. 1D).

## Understanding SAD with neuroimaging and computational data

SAD is commonly treated by three main approaches: pharmacological, psychological, or a combination of the two, depending on the individual's profile (Ströhle *et al*., 2018; Szuhany & Simon, 2022). These therapies take effect by altering the activity of key brain regions (e.g. measured by either resting state fMRI or task-based fMRI) implicated with the processing of social information. For example, pharmacological and psychotherapeutic forms of treatment lower amygdala activity in response to aversive stimuli (Goldin *et al*., 2013; Klumpp *et al*., 2013; Klumpp &. Fitzgerald, 2018), while cognitive-behavioural therapy (CBT) improves emotion regulation by increasing prefrontal and occipitotemporal activation (Brooks & Stein, 2015).

Neuroimaging methods, therefore, present an objective measure that can be used to determine the biological components underlying behavioural changes, assess responsivity to treatments, and generate predictions for health outcomes as 'neuromarkers' (Gabrieli *et al*., 2015). Resting-state fMRI (rs-fMRI), measuring intrinsic spontaneous fluctuations in blood-oxygen level-dependent signal, is a popular approach thanks to its ease of use in clinical practice (Fox & Greicius, 2010). In SAD, rs-fMRI has identified connectivity changes underlying clinical improvement after completion of a CBT program (Yuan *et al*., 2016, 2018) and, as a neuromarker, predicted treatment response to group CBT (Yuan *et al*., 2017). Task-based fMRI has further identified similar neurocognitive mechanisms underlying symptom reduction in SAD following common psychotherapies (Goldin *et al*., 2021). Although neuromarkers can accurately predict treatment response for SAD (e.g. Santos *et al*., 2019; Fig. 1C), this method also demonstrates low replicability (Ashar *et al*., 2021) reflecting issues concerning the reliability of fMRI measures (Noble *et al*., 2019) and the heterogeneity of mental health disorders (Forbes *et al*., 2023). As one example, significant within-group differences observed in precuneus and amygdala rsFC among individuals with SAD, in the absence of any group-level difference with healthy controls (Mizzi *et al*., 2024), further necessitates an approach accounting for patient heterogeneity when employing a predictive (neural) model (Talmon *et al*., 2021).

To this end, computational models provide a theory-driven and more nuanced measure of the latent cognitive processes shaping brain activity and behaviour (Karvelis *et al*., 2023). By establishing a 'computational phenotype', a set of parameters characterizing an individual's cognitive mechanisms (Patzelt *et al*., 2018), inter- and intra-individual differences—including responsivity to treatment—can be measured (Fig. 1B). Importantly, Koban *et al*. (2023) offer a framework in which computational modeling and fMRI can be used (either separately or together) to generate more specific and precise prediction models. In their study, individuals with SAD report lower activity of the FPN, correlating with reduced learning parameter from positive feedback and enhanced learning from negative feedback. Responsivity to treatment, behaviourally manifesting through changes in social learning rates, could therefore be inferred by a corresponding increase in FPN activity (Fig. 1D). This is reflected in a recent study where completion of a CBT program was found to normalize activity of the FPN among those with SAD (Haller *et al*., 2024). Furthermore, computational and neuroimaging data can be directly combined through a 'model-based fMRI' approach, in which brain regions associated with computational processes are identified by including estimates of latent variables as predictors of neural signals (e.g. Gläscher & O'Doherty, 2010; Zhang & Gläscher, 2020; Zhang *et al*., 2020; Katahira & Toyama, 2021). These computationally informed brain response patters could be used as complementary neuromarkers to assess treatment efficacy.

Informing patient-specific interventions through computational phenotyping is currently limited by the low reliability (Brown *et al*., 2020; Waltmann *et al*., 2022; Vrizzi *et al*., 2023) and poor psychometric properties (Karvelis *et al*., 2023) of certain computational measures. Furthermore, this approach requires knowledge regarding the influence of specific interventions to specific underlying mechanisms (Reiter *et al*., 2021; Berwian *et al*., 2023). However, progress is being made with determining sources of within-participant parameter variability (Schaaf *et al*., 2023) and mapping psychotherapy interventions to components of cognition and behaviour (Norbury *et al*., 2024). It is therefore important to determine the within-subject reliability of model parameters in healthy controls and individuals with SAD when informing the neurocomputational treatment of social anxiety.

## Conclusion

Computational psychiatry aims to accurately map the cognitive and mechanistic foundation underlying behavioural changes observed in mental health disorders. Emerging work has uncovered the neurocomputational mechanisms underlying biased processing of social feedback in social anxiety, an approach capturing patient heterogeneity. We advocate for future studies to investigate the potential for validated computational and cognitive models as a marker for treatment response.
